# Transient ischemia-reperfusion induces cortical hyperactivity and AMPAR trafficking in the somatosensory cortex

**DOI:** 10.18632/aging.102881

**Published:** 2020-03-09

**Authors:** Yuanyuan Li, Ran Ding, Feifei Wang, Cuiping Guo, Aili Liu, Liangpeng Wei, Shiyang Yuan, Feng Chen, Shaowei Hou, Zengguang Ma, Yan Zhang, Robert H. Cudmore, Xiaochuan Wang, Hui Shen

**Affiliations:** 1 School of Biomedical Engineering, Tianjin Medical University, Tianjin, China; 2 Chinese Institute for Brain Research, Beijing (CIBR), Beijing, China; 3 Department of Pathophysiology, School of Basic Medicine, Key Laboratory of Ministry of Education of China for Neurological Disorders, Tongji Medical College, Huazhong University of Science and Technology, Wuhan, China; 4 Tianjin Key Laboratory of Retinal Function and Diseases, Tianjin Medical University Eye Hospital, Eye Institute and School of Optometry and Ophthalmology, Tianjin Medical University, Tianjin, China; 5 Department of Physiology and Membrane Biology, University of California Davis School of Medicine, Sacramento, CA 95817, USA; 6 Division of Neurodegenerative Disorders, Co-innovation Center of Neuroregeneration, Nantong University, Nantong, China; 7 Research Institute of Neurology, General Hospital, Tianjin Medical University, Tianjin, China

**Keywords:** transient ischemia-reperfusion, somatosensory cortex, Ca transients ^2+^, AMPA receptor, dendritic spine

## Abstract

Brain ischemia results from cardiac arrest, stroke or head trauma. The structural basis of rescuing the synaptic impairment and cortical dysfunctions induced in the stage of ischemic-reperfusion can occur if therapeutic interventions are applied in time, but the functional basis for this resilience remains elusive. Here, we explore the changes in cortical activity and a-amino-3-hydroxy-5-methyl-4-isoxazole propionic acid receptor (AMPAR) GluA1 subunit in spine (sGluA1) after transient ischemia-reperfusion *in vivo* for 28 days. Using *in vivo* two-photon microscopy in the mouse somatosensory cortex, we found that the average frequency of Ca^2+^ transients in the spine (there was an unusual synchrony) was higher after 15 min of ischemia-reperfusion. In addition, the transient ischemia-reperfusion caused a reflective enhancement of AMPARs, which eventually restored to normal. The cortical hyperactivity (Ca^2+^ transients) and the increase in AMPARs were successfully blocked by an NMDA receptor antagonist. Thus, the increase of AMPARs, cortical hyperactivity and the unusual synchrony might be the reason for reperfusion injury after short-term transient ischemia.

## INTRODUCTION

Compared to other tissues and organs in the body, the brain is particularly vulnerable to ischemic injury. A transient period of cerebral ischemia induces selective and delayed neuronal cell death [1] as well as a rapid and sustained structural reorganization of dendritic spines [2]. Previous work implicated a variety of different mechanisms in the demise of neurons and their synaptic networks during ischemia, including ischemic depolarization, excitotoxic [Ca^2+^]i changes, non-selective ion channels, membrane breakdown, and reperfusion injury [3–6]. Although many mechanistic data exist for simulated stroke *in vitro*, it is unclear whether the same mechanisms are at work *in vivo*, or how these events are related to the structural and functional disruption of synaptic networks.

Glutamate is the major excitatory neurotransmitter in the central nervous system (CNS) and plays a key role in maintaining normal physiological processes. The a-amino-3-hydroxy-5-methyl-4-isoxazole propionic acid receptor (AMPAR) and N-methyl-D-aspartic acid receptor (NMDAR) are the main excitatory glutamate receptors. Ischemia initiates a massive release of the excitatory transmitter glutamate and provokes further depolarization due to activation of AMPARs and NMDARs as well as voltage-gated Ca^2+^ channels. Accumulating evidence suggests that extrasynaptic NMDARs play a unique role in cell death [7–9]. Extrasynaptic receptors may become activated when excessive levels of glutamate spill out of the synapse during prolonged depolarization. This factor could be considered to initiate signaling cascades that are uniquely activated by extrasynaptic receptors [10, 11]. There are many reports on the neurotoxic properties of glutamate connected with the activation of AMPAR, which leads to changes in the permeability of the postsynaptic membrane for monovalent ions (sodium and potassium), the enhancement of sodium influx, and short-term depolarization of the postsynaptic membrane [12]. This in turn leads to enhancement of calcium influx into cells via both agonist-dependent and potential-dependent channels [3, 13, 14]. Although the restored dendritic structures remain plastic to rebuild the cortical network [2] and activity-dependent changes in AMPAR [15] after ischemia-reperfusion have been examined, the trafficking of AMPAR *in vivo* during ischemia-reperfusion in real time has not been previously studied.

In the present study, reversible global cerebral ischemia model combined with long-term two-photon calcium imaging and transfecting layer 2/3 pyramidal neurons in somatosensory in utero electroporation were used to investigate the changes in cortical activity and AMPAR trafficking about transient ischemia-reperfusion injury. We detected that the average frequency of Ca^2+^ transients in the spine of the somatosensory cortex was higher after ischemia. Moreover, transient ischemia can cause a reflective enhancement of AMPAR. The cortical hyperactivity and the increase in sGluA1 were successfully blocked by an NMDAR antagonist.

## RESULTS

### Transient ischemia-reperfusion induces cortical hyperactivity

**Figure 1:**
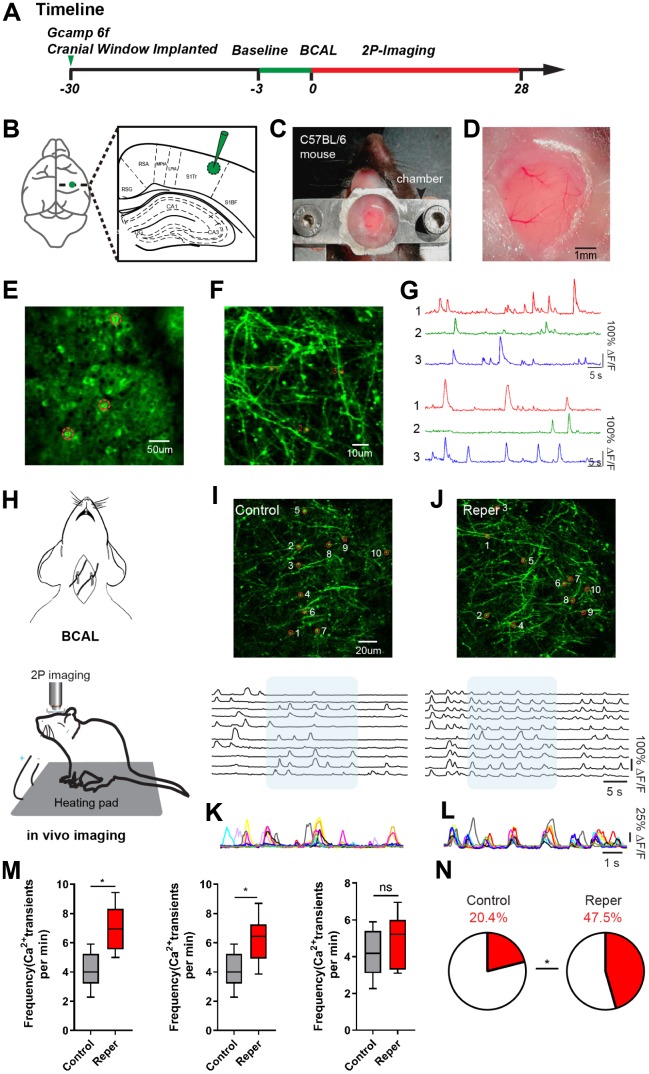
**Transient ischemia-reperfusion induces cortical hyperactivity.** (**A**) Timeline of the experiment. Green arrow, virus injection time; the green line and red line indicate the two-photon imaging time course. (**B**) Virus injection sites. (**C**) Chamber for chronic two-photon imaging. (**D**) Picture of the craniotomy window. (**E, F**) Layer 2/3 cortical neurons and layer1 spines images. The red circle represents the selected somata (**E**) and spine (**F**). (**G**) The spontaneous Ca^2+^ transients of neurons and spines in (**E, F**) maps. (**H**) Experimental methods for inducing ischemia and for *in vivo* imaging. (**I, J**) Top, layer 1 (30~50 um) spines imaged *in vivo* on the 3rd day after reperfusion, where the red circle represents the selected spine in control (**I**) and Reper (**J**) mice. Bottom, the traces of selected spines. (**K, L**) Superimposed traces from the shaded areas in i and j. Each color represents a different cell. (**M**) The average frequencies of Ca^2+^ transients in control and Reper mice. The 3rd hour (left, n=395 spines in 5 controlmice, 438 spines in 6 Reper mice), the 3rd day (middle, n= 405 spines in 5 control mice, 432 spines in 6 Reper mice) and the 14^th^ day (right, n= 397 spines in 5 control mice, 436 spines in 6 Reper mice). (**N**) The fractions of hyperactive spines in control mice (n= 405 spines) and Reper mice (n= 432 spines) on the 3rd day after reperfusion. *P < 0.05, ns, not significant, Student’s t-test. Error bars = s.e.m.

To observe the changes in cortical activity after transient ischemia-reperfusion, we injected ultra-sensitive protein calcium sensor GCaMP6f [16] into the layer 2/3 of the somatosensory cortex (Figure 1B). A chronic cranial window [17, 18] on the somatosensory cortex was established (Figure 1C, 1D). Following about 4-week, we used two-photon calcium imaging to characterize the activity levels of the spine populations (in layer 1, 30~50 um) and populations (in layer 2/3) (Figure 1E–1G) which were stably expressed a GCaMP6f over 28-day (Figure 1A). Then we examined the effects of transient global ischemia on spine activity levels by ligating the bilateral common carotid arteries (BCAL) (Figure 1H). In the somatosensory cortex, where ~99% reduction in blood flow was induced (Supplementary Figure 1A, 1B), we observed a wave like cortical spreading depolarization or depression (CSD) [19], which nearly complete depolarization of brain cells and silenced brain activity until reperfusion (Supplementary Figure 1B, 1C). The blood flow was restored to the pre-ischemia level within 3 min after reperfusion (Supplementary Figure 1D, reperfusion 3 min). We observed that dendrites became beaded and spines were distorted within 10-15 min after ligation (Supplementary Figure 1E, 1F).

Moreover we observed an aggravation of spine dysfunction. This aggravation manifested itself in several ways. First, we found that in a subset of treated mice (71%), the increased hyperactivity was associated with an unusual synchrony (Figure 1I–1L). Second, both at the 3rd hour and on the 3rd day, the average frequency of Ca^2+^ transients markedly increased in ischemia-reperfusion (Reper) mice compared with controls. The average frequency of Ca^2+^ transients recovered to the pre-stroke level 14 days after transient ischemia-reperfusion (Figure 1M). Finally, the proportion of hyperactive (>6 transients per min) spines was two- to three-fold larger in Reper mice than in control mice (Figure 1N).

### NMDAR NR2B antagonist rescues cortical hyperactivity and the unusual synchrony

**Figure 2:**
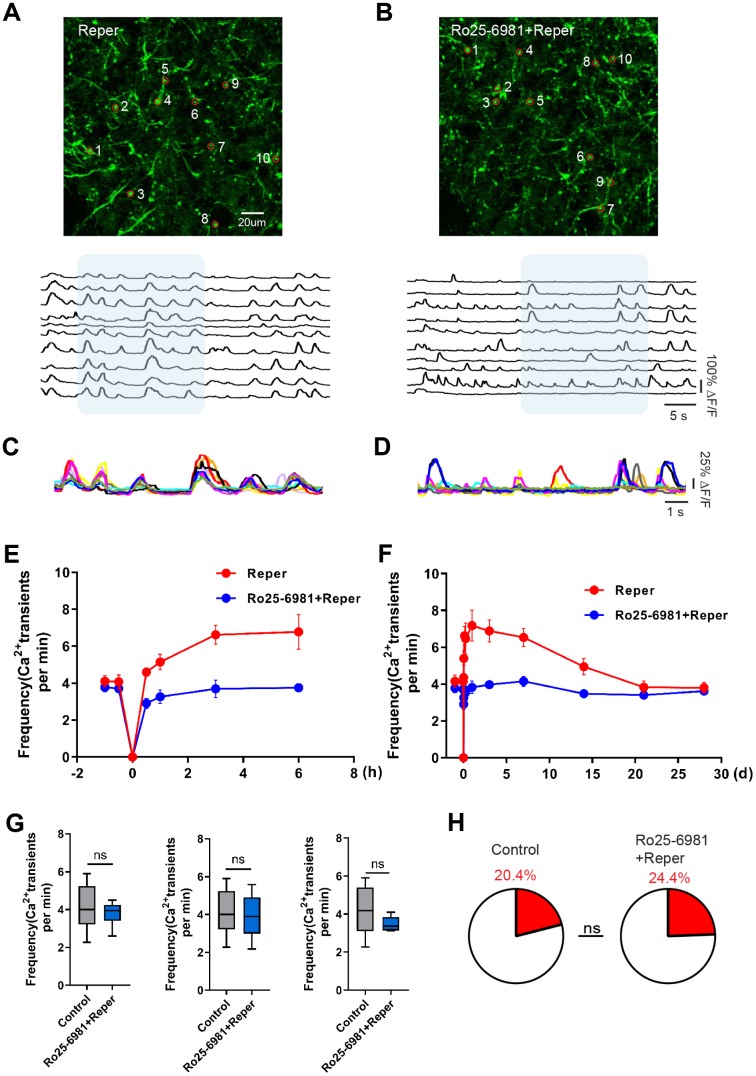
**Transient global cerebral ischemia-reperfusion aggravates NMDAR-dependent spine dysfunction.** (**A, B**) Top, layer 1 (30~50 um) spines images on the 3rd day after reperfusion, where the red circle represents the selected spine in Reper (**A**) and Ro25-6981-treated (**B**) mice. Bottom, the traces of selected spines. (**C, D**) Superimposed traces from the shaded areas in a and b. Each color represents a different cell. (**E, F**) The overall trend of the average frequency of Ca^2+^ transients before and after 6 hours (**E**) and 28 days (**F**) of ischemia-reperfusion. (**G**) The average frequency of Ca^2+^ transients in Reper and Ro25-698-treated mice. The 3rd hour (left, n= 438 spines in 6 Reper mice, n= 312 spines in 4 Ro25-698-treated mice), the 3rd day (middle, n= 432 spines in 6 Reper mice, n= 328 spines in 4 Ro25-698-treated mice) and the 14^th^ day (right, n=436 spines in 6 Reper mice, n= 329 spines in 4 Ro25-6981-treated mice). (**H**) The fractions of hyperactive spines Reper mice (n= 432 spines) and Ro25-698-treated mice (n=328 spines) on the 3rd day after reperfusion. *P < 0.05, ns, not significant, Student’s t-test. Error bars = s.e.m.

**Figure 3:**
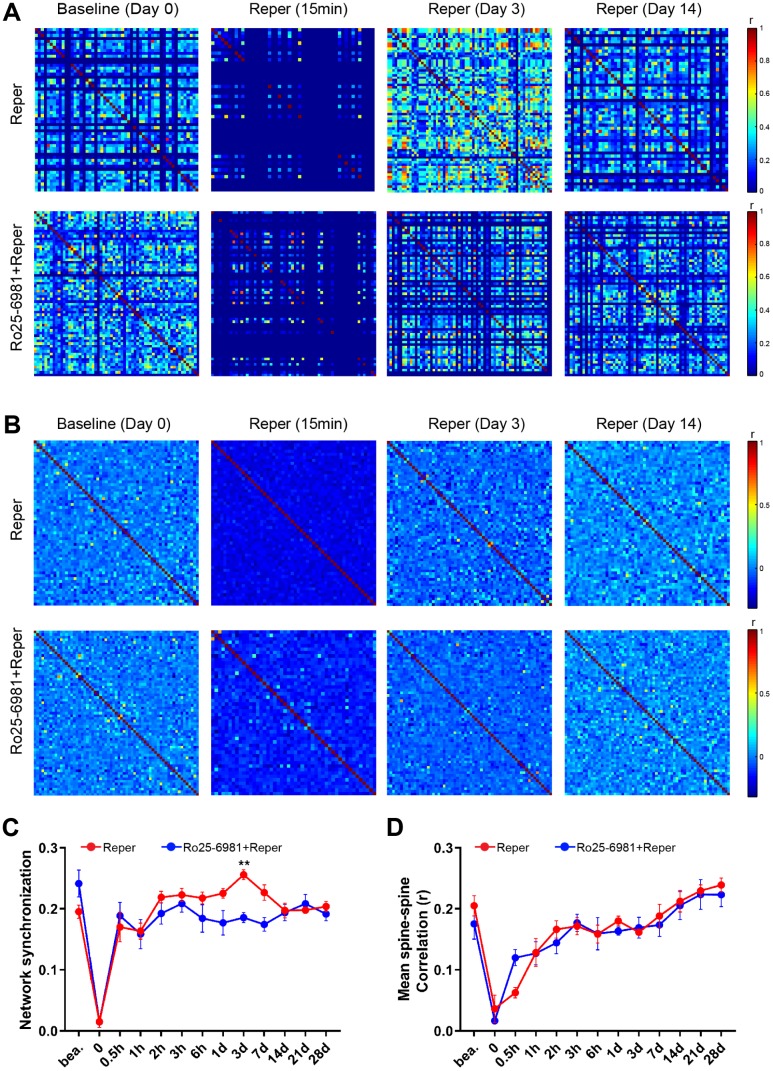
**NMDA receptor blockade rescues BCAL induced network synchronization but not microcircuit dysfunction.** (**A**) Raster plots depicting changes in activity (ΔF/F) over time for a representative correlation matrices quantifying network synchronization between each spine and every other (**B**) Representative correlation matrices quantifying functional connectivity between each spine and every other spine. (**C, D**) The in network synchronization and mean spine-spine correlation on the 3rd day after reperfusion. n= 1044 spines in 6 Reper mice, n= 824 spines in 4 Ro25-6981-treated mice. **P < 0.01, two-way ANOVA with Bonferroni correction. Error bars = s.e.m.

The activation of single excitatory synapses causes calcium accumulations in individual dendritic spines, mediated by NMDARs [20, 21], which can be imaged to measure the tuning of single synapses *in vivo* [21, 22]. Next, we wondered whether NMDA receptor antagonist treatment could ameliorate neuronal dysfunction at an earlier disease stage. There are evidence supports the notion that the activation of NR2A-containing NMDARs promotes neuroprotection, while the activation of NR2B-containing NMDARs results in excitotoxicity [23]. Hence we intraperitoneally injected mice with the NR2B antagonist Ro25-6981(TOCRIS #1594) at 10 mg/kg, 30-45 minutes prior to BCAL and then once a day for 3 days. After Ro25-6981 exposure, we found the unusual synchrony of spines were ameliorated (Figure 2A–2D) and the average frequency of Ca^2+^ transients significantly reduced (Figure 2E–2G). Furthermore, the proportion of hyperactive spines was almost restored to baseline levels (Figure 2H). In addition, the network synchronization significantly increased on the 3rd day after ischemia-reperfusion within somatosensory cortex microcircuits. Ro25-6981 treatment rescued the network synchronization of spines (Figure 3A, 3C). Surprisingly, BCAL did not alter the functional connectivity within the microcircuits (Figure 3B, 3D). Such synchrony, which has not been observed in WT mice and is seen only in Reper mice, may underlie reperfusion damage following transient ischemia-reperfusion. (Figure 3A, 3C). Together, these results demonstrate that transient global ischemia leads to NMDAR-dependent cortical hyperactivity and an unusual synchrony.

### Imaging and electrophysiological properties of cortical neurons with tagged AMPARs *in vivo*

**Figure 4:**
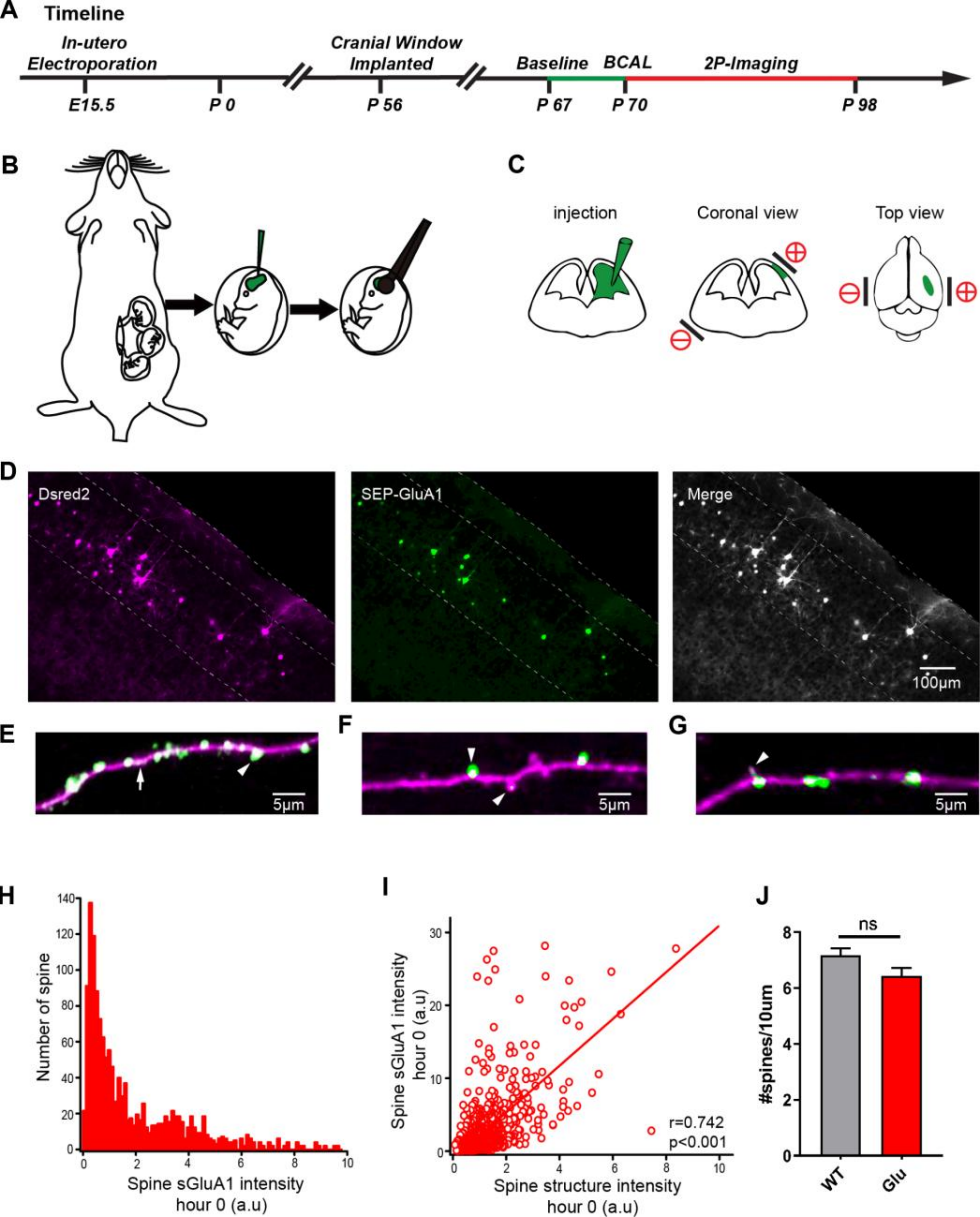
**Expression of SEP-GluA1 in layer 2/3 somatosensory cortex neurons *in vivo*.** (**A**) Timeline of experimental design. The green line and the red line indicates the two-photon imaging time course. (**B, C**) Schematic drawing of in utero electroporation. +/- means positive and negative polar, respectively. (**D**) Representative images showing expression of dsRed2 (purple), SEP-GluA1 (green) and their overlap (white). (**E**–**G**) SEP-GluA1 in green, dsRed2 in magenta, and their overlap in white. (**H**) Histogram of spine sGluA1 intensity before BCAL at hour 0. (**I**) Correlation between spine sGluA1 and spine size before BCAL at hour 0. n= 1381 spines. *r*, Pearson's linear correlation coefficient, *p* value is from Monte-Carlo shuffling. (**J**) Quantification of spine density. n= 27 neurons in 5 control mice, 24 neurons in 5 Glu mice. ns, not significant, Student’s t-test. Error bars = s.e.m.

Many reports have shown that overactivation of the AMPAR can induce short-term depolarization of the postsynaptic membrane [12], which in turn can lead to enhancement of calcium influx into cells via both agonist-dependent and voltage-gated ion channels [3, 13, 14]. To monitor AMPAR dynamics and spine turnover in the somatosensory cortex, E15.5 mouse embryos were electroporated in-utero with the AMPAR GluA1 subunit tagged with a pH-sensitive form of GFP (Super Ecliptic pHluorin, SEP), the AMPAR GluA2 subunit tagged with myc, and a morphological marker dsRed2 (Figure 4A–4C). Two-photon imaging of GluA1 in brain slices of electroporated mice show that the transfected neurons have only modest overexpression of GluA1 (Figure 4D). We then visualized the AMPARs by means of two-photon imaging *in vivo* (Supplementary Figure 2A, 2B). The images of apical dendrites from layer 2/3 neurons in both channels were bright signals (Supplementary Figure 2C). The transfected neurons had uniform expression of dsRed2 throughout the cells and had a high expression of SEP-GluA1 in the synaptic spines with a relatively lower expression in the dendritic shafts (Figure 4E) consistent with previous findings [24]. Moreover, sGluA1 expression was dramatically different in different spines along the same dendrite within a few microns of each other (Figure 4F) and even within immature filopodia-like structures [25] (Figure 4G). The sGluA1expression confirmed that the resolution of our system was sufficient to track AMPAR *in vivo*. The basal expression of SEP- GluA1 in spines *in vivo* had a wide distribution and was correlated with spine size (Figure 4H, 4I). There was no difference in spine density between the groups (Figure 4J). Stable images were obtained over a 28-day period with no evidence of photo-bleaching (Supplementary Figure 2D). These results show that fluorescent protein-tagged AMPARs can be visualized *in vivo* and have no effect on spine density, which is correlated with the number of postsynaptic AMPARs [26, 27] and is a determinant of synaptic strength [28]. In addition, we performed two-photon targeted patching as previously mentioned in the transfected mouse group [29] and two-photon shadow patching in the control mouse group [30] with no pre-labeled neurons. We found that the active and passive electrophysiological properties of the Glu neurons were all similar to those of the control group (Supplementary Figure 3) and the tagged AMPARs have no effect in excitatory and inhibitory synaptic transmission (Supplementary Figure 4).

### AMPAR dynamics appears in transient ischemia-reperfusion

**Figure 5:**
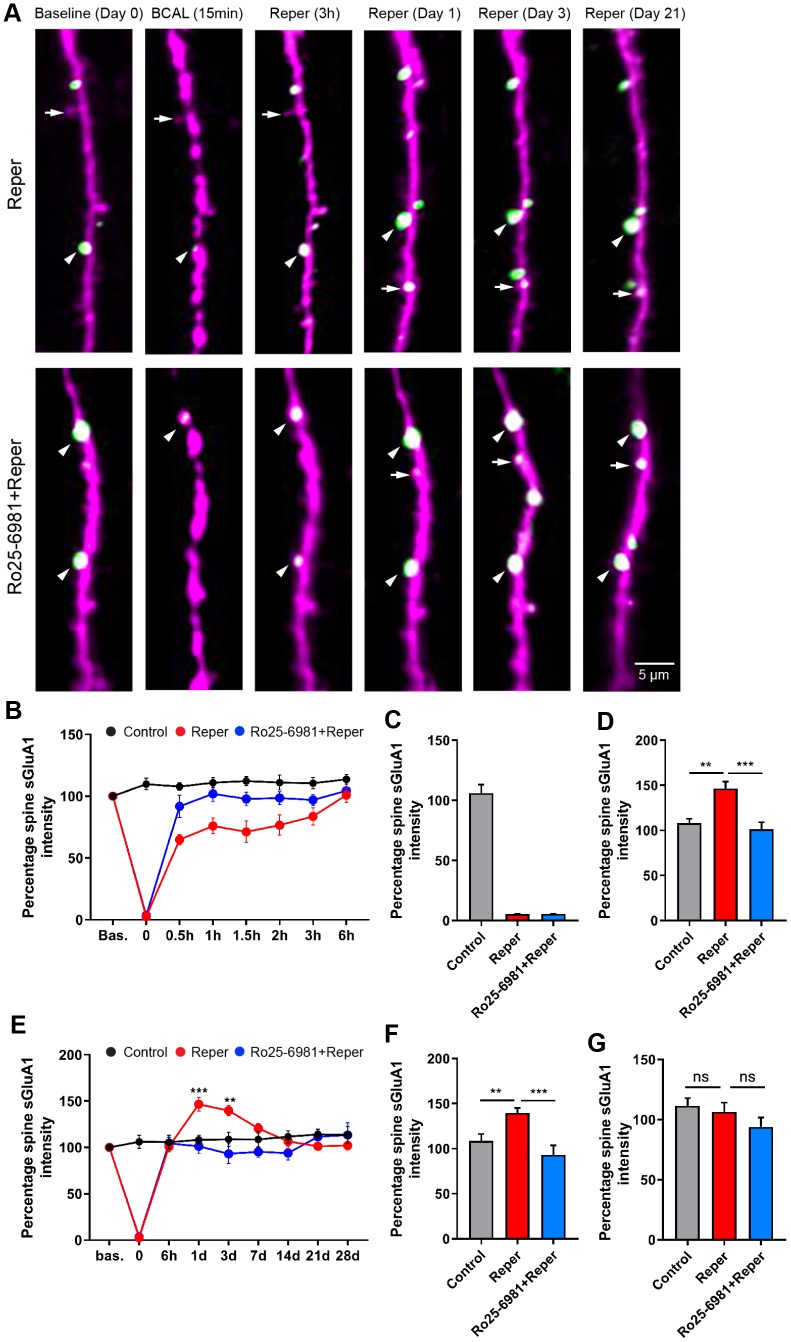
**BCAL leads to an NMDAR-dependent increase in spine sGluA1 *in vivo* in apical dendrites of layer 2/3 neurons in the somatosensory cortex**. (**A**) Representative images of spines on layer 2/3 apical dendrites in Reper or Ro25-6981 treated mice. Arrowheads indicate stable spines and arrows mark unstable spines, including new spines and eliminated spines. (**B, E**) The overall trend of the Spine sGluA1 intensity in control, Reper and Ro25-6981-treated mice before and after 6 hours (**B**) and 28 days (**E**) of ischemia-reperfusion. **P < 0.01, ***P < 0.001, two-way ANOVA with Bonferroni correction. Error bars= s.e.m. (**C**–**G**) Percentage spine sGluA1 at the beginning of reperfusion (**C**), on the first day (**D**, n= 425 spines in 5 control mice, n= 379 spines in Reper mice, and n= 411 spines in 5 Ro25-6981-treated mice.), the 3rd day (**F**, n= 410 spines in 5 control mice, n= 283 spines in Reper mice, and n= 274 spines in 5 Ro25-6981-treated mice.) and the 14^th^ day (**G**, n= 410 spines in 5 control mice, n= 283 spines in Reper mice, and n= 274 spines in 5 Ro25-6981-treated mice.) following ischemia-reperfusion. **P < 0.01, ***P < 0.001, ns, not significant, one-way ANOVA with Bonferroni correction. Error bars = s.e.m.

The spine AMPAR content is a strong correlate of synaptic strength and plays a critical but functionally contradictory role in the pathophysiology of stroke [31–33], yet no previous studies have investigated *in vivo* AMPAR trafficking in layer 2/3 pyramidal neurons after transient ischemia/reperfusion. We first examined the effects of transient global ischemia, and found that dendrites became beaded, spines were distorted and spine sGluA1 greatly reduced during ischemia (Figure 5A–5C and Supplementary Figure 5A). A previous study has suggested a decrease in pH following ischemia [34, 35]. To evaluate whether pH is associated with the change in spine sGluA1, we performed western blot experiments to analyze the AMPAR levels 15 min after ischemia, and found that neither the total expression nor the surface expression of GluA2 significantly changed in BCAL mice compared with control mice (Supplementary Figure 6A–6D). The total expression of GluA1 did not change (Supplementary Figure 6A, 6F), while there was a significant reduction in the surface expression of GluA1 (Supplementary Figure 6D, 6E). These results indicated that ischemia led to a decrease in spine sGluA1. After ischemia, the beaded dendrites rapidly recovered, and the majority of spines were restored when blood vessels were re-perfused [2]. We further monitored surface AMPAR dynamics on the stability of synaptic structure over 28 days *in vivo*, and found that ischemia/reperfusion led to an ~99% decrease in spine sGluA1 on pre-existing spines after transient ischemia-reperfusion. This decrease in spine sGluA1 following BCAL was rapid and persisted for at least 6 hours (Figure 5B). In contrast, we observed a significant increase in average spine sGluA1 on the first day (Figure 5D) and the 3rd day (Figure 5F) after transient ischemia-reperfusion. However, the intensity of spine sGluA1 recovered to the pre-stroke level on the 7th day (Figure 5G). Taken together, these results suggest that transient ischemia can cause rapid substantial damage to AMPARs and then reflective enhancement, but these changes can be largely restored following reperfusion.

**Figure 6:**
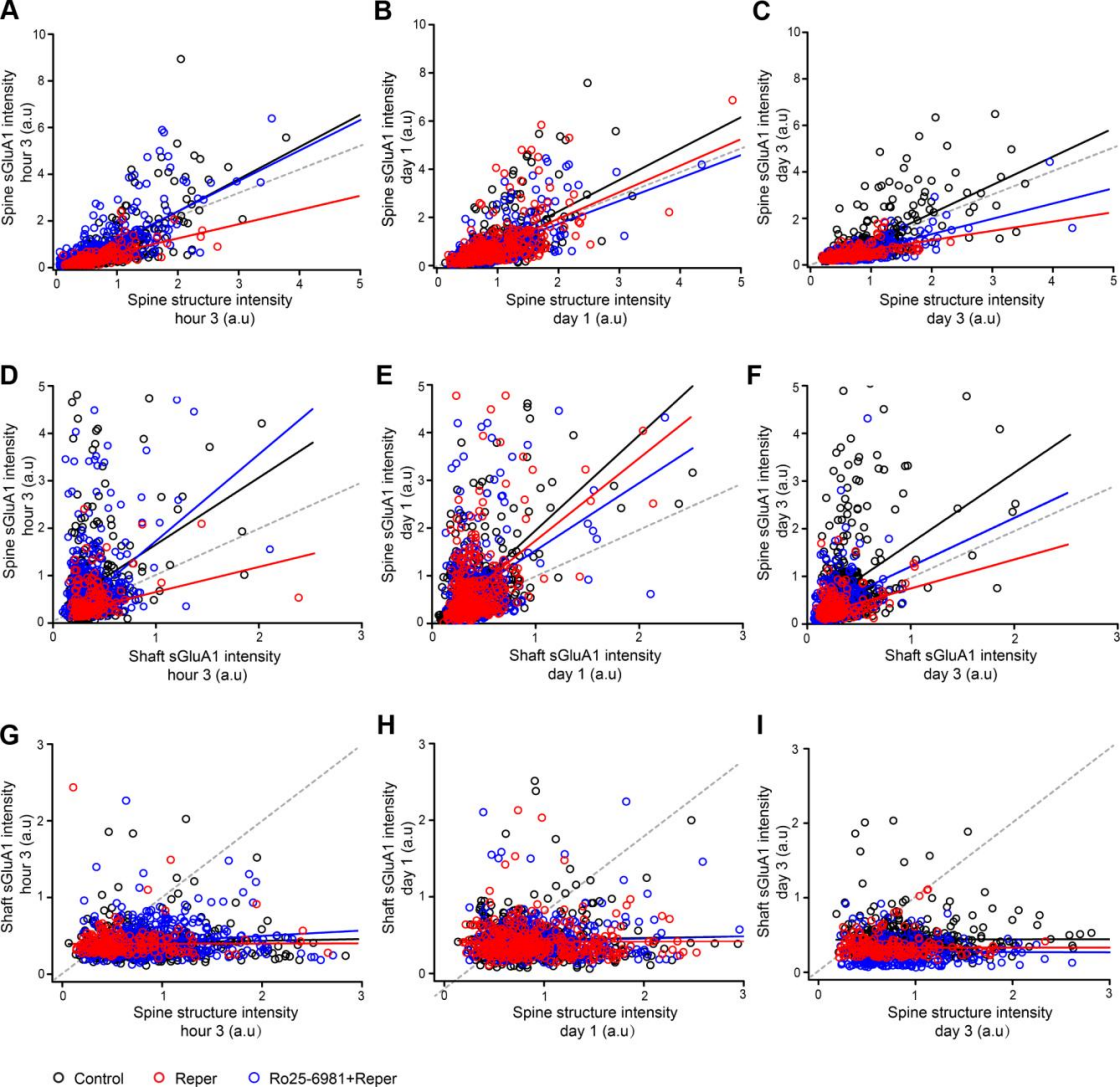
**Ro25-6981 rescues the changes in spine sGluA1 with shaft sGluA1 and spine size following BCAL.** (**A**–**C**) Correlation between spine sGluA1 intensity and spine structure intensity at the 3rd hour (**A**), on the first day (**B**) and the 3rd day (**C**) of ischemia-reperfusion in control, Reper and Ro25-6981-treated mice. (**D**–**F**) Correlation between spine sGluA1 intensity and shaft sGluA1 intensity at the 3rd hour (**D**), on the first day (**E**) and the 3rd day (**F**) of ischemia-reperfusion in control, Reper and Ro25-6981-treated mice. (**G**–**I**) Correlation between shaft sGluA1 intensity and spine structure intensity at the 3rd hour (**G**), on the first day (**H**) and the 3rd day (**I**) of ischemia-reperfusion in control, Reper and Ro25-6981-treated mice. The 3rd hour n= 342 spines in 5 control mice, n= 418 spines in Reper mice, and n= 457 spines in 5 Ro25-6981-treated mice. The first day n= 425 spines in 5 control mice, n= 379 spines in Reper mice, and n= 411 spines in 5 Ro25-6981-treated mice. The 3rd day n= 410 spines in 5 control mice, n=283 spines in Reper mice, n= 274 spines in 5 Ro25-6981-treated mice. *r*, Pearson's linear correlation coefficient. *p*, Pearson's correlation t test.

To further characterize the changes in spine sGluA1 following ischemia-reperfusion, we investigated the correlations between spine size, spine sGluA1 and shaft sGluA1. We observed a positive correlation between spine sGluA1 intensity and spine size in both the control and the Reper group after reperfusion (Figure 6A–6C). Interestingly, the slope of Reper group on the first day (Figure 6B, slope= 1.1) is larger than that of the 3rd hour (Figure 6A, slope= 0.6) and the 3rd day (Figure 6C, slope= 0.4). These results indicate that while changes in spine size and sGluA1 intensity are correlated, the decrease in spine sGluA1 density is larger. This observation is consistent with our result that the total average spine sGluA1 significantly decreased following ischemia-reperfusion (Figure 5B, 5C). However, our data suggest that the intensity of spine sGluA1 rapidly recovers and increases on the first day and the 3rd day after ischemia-reperfusion (Figure 5D, 5F), therefore we further assessed the correlations between spine sGluA1 intensity and shaft sGluA1 intensity. We observed a positive correlation between spine sGluA1 intensity and adjacent shaft sGluA1 intensity on the first day after reperfusion (Figure 6E, *r* = 0.44). Moreover, the slope of Reper group (Figure 6E, slope= 1.73) was larger than that both at the 3rd hour (Figure 6D, slope= 0.53) and on the 3rd day (Figure 6F, slope= 0.51). In addition, the shaft sGluA1 intensity and spine size were not correlated after reperfusion (Figure 6G, 6H). Taken together, these results demonstrate there is a much larger increase in spine sGluA1 than in spine size. Furthermore, initial extrasynaptic (shaft) sGluA1 insertion may supply GluA1 for spine incorporation on the first day after reperfusion. Although spine incorporation of GluA1 is ongoing, initial extrasynaptic (shaft) sGluA1 insertion ceases. These results may lead to spine sGluA1 content recovery to the pre-stroke level on the 7^th^ day (Figure 5G).

### Increases in the level of spine sGluA1 after ischemia-reperfusion is NMDA receptor dependent

To test whether the ischemia-reperfusion evoked increase in spine sGluA1 was NMDAR dependent, we first intraperitoneally injected mice with the NR2B antagonist Ro25-6981 at 10 mg/kg, 30-45 minutes prior to the control mice and the BCAL mice, then once a day for 3 days. After Ro25-6981 exposure, we found no change (Supplementary Figure 5D) in spine sGluA1 over 28 days. In addition, there was no change in spine size, spine sGluA1 or shaft sGluA1 in mice treated with Ro 25-6981 compared with the control mice at the 3rd hour (Supplementary Figure 5B) and on the 3rd day (Supplementary Figure 5C). Remarkably, Ro25-698 application completely blocked the increase in spine sGluA1 following ischemia-reperfusion (Figure 5A–5F), and the sGluA1 eliminated during BCAL can be blocked (Supplementary Figure 6E). Because of the decrease in pH following ischemia, we did not observe that Ro 25-6981 blocked the decrease in spine sGluA1 content during BCAL *in vivo* (Figure 5A–5C). These results suggest that the reflective enhancement of spine sGluA1 is an NMDA-receptor dependent process.

How do the correlations in spine size, spine sGluA1 and shaft sGluA1 changes after Ro25-6981 exposure? We determined the slope of the spine sGluA1 intensity and spine size (Figure 6A, slope=1.45), and the slope of the spine sGluA1 intensity and the shaft sGluA1 intensity (Figure 6D, slope=1.8). Both of them recovered to pre-stroke levels at the 3rd hour after reperfusion. These results are consistent with our results that the total average spine sGluA1 significantly increases following Ro25-698 exposure (Figure 5). In addition, we observed that the correlation between spine sGluA1 intensity and adjacent shaft sGluA1 intensity on the first day after Ro25-6981 exposure recovered to 0.39 (Figure 6E), and the slope was 1.41. This indicated that Ro25-6981 exposure could block the initial extrasynaptic (shaft) sGluA1 insertion and the GluA1 for spine incorporation. Taken together, these results demonstrate that ischemia-reperfusion leads to a long-lasting spine surface incorporation of GluA1, which is NMDAR-dependent.

## DISCUSSION

Our study investigated the changes in cortical activity and found significant increases in spine and neuronal activity levels. Moreover, the number of pathologically hyperactive spines increased and the increased hyperactivity was associated with an atypical synchrony. The long-lasting neuronal hyperexcitability in the periinfarct cortex was observed after ischemia-reperfusion [36–39], peaking 4 weeks after stroke and persisting for 60 days [37, 40]. These times did not correlate well with the temporal profile of the functional rewiring we observed in layer 2/3 somatosensory neurons after ischemia-reperfusion. We observed that the average frequency of Ca^2+^ transients recovered to the pre-stroke level at approximately 14 days after transient ischemia-reperfusion. Bilateral common carotid artery ligation (BCAL) of 15 min did not cause the infarct area. The neurons survived the initial insult, which similar to the penumbra population, may potentiate subthreshold activity and cause action potential firing, perhaps rendering the neurons more responsive to the signals [40]. Pathophysiological increases in glutamate can be measured in patients during and after stroke. The glutamate spilling out of the synapse could initiate signaling cascades that are uniquely activated by extrasynaptic receptors [10, 11]. Therefore, we injected mice with the NMDAR NR2B antagonist and found that it spared the hyperactivity and the abnormal synchrony of cortical activity, which was otherwise attenuated by reperfusion.

In most previous studies, spine structural dynamics have been used as a measure of synaptic plasticity. However, when the spine turnover was not significantly different, changes in AMPAR expression were observed between stimulated and control mice [2]. The results suggest that the key events might be missed if we investigated spine turnover in isolation. Spine AMPAR content is a strong correlate of synaptic strength [31–33], although the level of spine AMPARs does not directly measure synaptic strength. Moreover, AMPAR function plays a critical but functionally contradictory role in the pathophysiology of ischemia-reperfusion. Because the long-term dynamic correlation between AMPAR expression and spine properties have not been investigated after ischemia-reperfusion *in vivo*, this is the primary goal of the study. Our study visualizes AMPARs *in vivo* in awake mice and the real time dynamic expression of AMPARs and we demonstrate that spine sGluA1 is greatly reduced during ischemia. Under ischemic conditions, the trafficking of these AMPARs from the surface to the cytoplasm, directly affects their function in excitotoxicity and related neurological diseases [41, 42]. However, after transient ischemia-reperfusion, we observed a significant increase in average spine sGluA1 on the first day and on the 3rd day. Other reports suggest that ischemia-reperfusion injury releases inflammatory factors such as glial-derived tumor necrosis factor (TNF), enhances trafficking of glutamate receptors onto post-synaptic membranes, and improves presynaptic neurotransmitter release of glypican [43]. Then, glypican increases the number of AMPARs at postsynaptic sites [44]. Taken together, overactivation of the APMAR can induce severe neuronal damage and infarct evolution [45].

**Figure 7:**
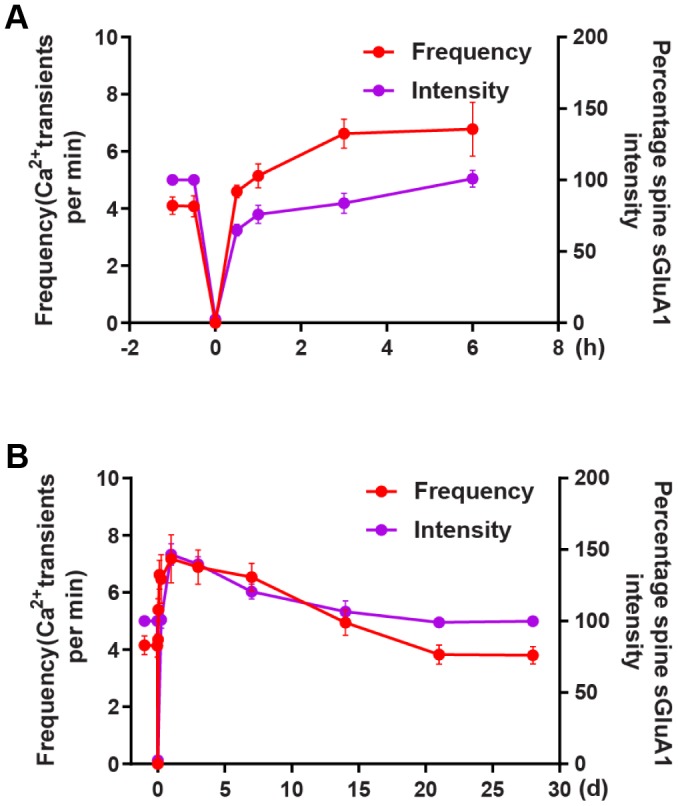
**The correlation between changes in Ca^2+^ transients and changes in AMPAR intensity after transient global ischemia-reperfusion.** (**A**) The overall trend of the average frequency of Ca^2+^ transients and the Spine sGluA1 intensity (SEP-GluA1 signal) before and after 6 hours of ischemia-reperfusion. (**B**) The overall trend of the average frequency of Ca^2+^ transients and the Spine sGluA1 intensity (SEP-GluA1 signal) before and after 28 days of ischemia-reperfusion.

There is a much larger increase in spine AMPARs both on the first day and on the 3rd day after ischemia-reperfusion. Increases in spine AMPARs on the first day after reperfusion are well coordinated with and distinctly from changes in shaft AMPARs. These shaft AMPARs may serve as a pool of extrasynaptic AMPARs for synaptic recruitment after reperfusion. The initial extrasynaptic (shaft) AMPARs insertion ceases may lead to the recovery of spine AMPARs to the pre-stroke level on the 7^th^ day after ischemia. We observed that the average frequency of Ca^2+^ transients reached a maximum approximately on the 5^th^ day and then recovered to the pre-stroke level approximately on the 14^th^ day after transient ischemia-reperfusion. In addition, we observed the increase time in Ca^2+^ signal was earlier than that in AMPAR during the first 6 hours (Figure 7A), while the decrease in AMPAR was faster than in the Ca^2+^ signals (Figure 7B). These results suggest that at the early stage Ca^2+^ transient increases and this leads to AMPAR increase, then AMPAR decrease as Ca^2+^ transient decrease. In the mammalian central nervous system, an overactivation of the AMPARs can lead to short-term depolarization of the postsynaptic membrane [12], and this in turn can lead to the enhancement of calcium influx into cells via both agonist-dependent and potential-dependent channels [3, 13, 14]. These data indicate that there is an articulation point within the first several days after ischemia-reperfusion where AMPARs switch from promoting neuronal death to promoting behavioral recovery.

In conclusion, our data suggest that the increase of AMPAR in the spines, cortical hyperactivity and the atypical synchrony may underlie reperfusion injury after short-term transient ischemia-reperfusion. The use of a glutamate receptor antagonist during the first several days after reperfusion can rescue the damage to synaptic structures and cortical function.

## MATERIALS AND METHODS

### Mice

C57BL/6 mice (8-10 weeks, 20-22 g) were purchased from the institute of zoology, Chinese academy of sciences. All mice were free access to food and water and maintained in a temperature and humidity controlled room with a reverse light:dark cycle (12:12) (All mice were bred in the Tianjin medical university of China-approved animal facility). All experiments were approved by the Animal Care and Use Committee of Tianjin Medical University, in compliance with National Institutes of Health guidelines.

### Stereotaxic virus injection

Mice (56-60 days old) were anesthetized with an isoflurane-oxygen mixture (1.5% vol isoflurane/vol O_2_) and given the analgesic buprenorphine (SC, 0.3 mg/kg). Virus injection was performed using a glass pipette beveled at 45° with a 15-20 μm opening and back-filled with mineral oil. A fitted plunger controlled by a hydraulic manipulator (Narashige, MO10) was inserted into the pipette and used to load and inject the viral solution. For calcium imaging with GCaMP6f, 20~30 nl of AAV2/1-syn-GCaMP6f-WPRE-SV40-containing solution (~2 × 10^13^ infectious units/ml) was slowly injected into somatosensory cortex (-1.5mm from bregma and 2.00mm from midline). To prevent backflow during withdrawal, the pipette was kept in the brain for over 15 min and then the plunger was withdrawn (~1 nl in volume) before the pipette was pulled up.

### In utero electroporation

Layer 2/3 progenitor neurons were transfected by in utero electroporation in E15.5 embryos as described previously [46]. The uterine horns were exposed and approximately 1 ul of buffer solution containing 1.2 ug/ul SEP-GluA1 and myc-GluA2 plasmid, 0.3 ug/ul Dsred2 plasmid (The SEP-GluA1, myc-GluA2 and Dsred2 plasmids were obtained from Johns Hopkins University School of Medicine [24] and a trace of Fast Green (Sigma) was pressure injected through a pulled-glass pipette into the right lateral ventricle of each embryo. Five pulses of 35 V for E15 (50 ms on, 950 ms off, 1 HZ) were delivered, targeting the somatosensory cortex, using 3 mm tweezer electrodes connected to a square wave electroporator (CUY21EDIT, π Protech).

### Global cerebral ischemia model

Reversible global cerebral ischemia was induced using the bilateral common carotid artery ligation (BCAL) model. Mice were anesthetized with an isoflurane-oxygen mixture (1.5% vol isoflurane/vol O_2_). The bilateral common carotid arteries and nerves were exposed and the bilateral common carotid arteries were separated from the carotid sheath. The mouse was subjected to 15 min of transient ischemia without anesthesia, and then the sutures were untied to reperfusion. When blood flow was reduced by >90% the BCAL model was considered successful, and the reperfusion of blood flow was confirmed in all ischemia mice. The control mice with a cranial window received the same surgical exposure of bilateral common carotid arteries except for the ligation step.

### Two-photon imaging

All imaging was performed with a two-photo microscope, using a Ti: Sapphire laser (model “Mai-Tai Deep See”, Spectra Physics). A Nikon water-immersion objective (25X, 1.10NA) was used. For calcium imaging experiments, the excitation wavelength was set to 920nm with 30 to 40 mW. SEP-GluA1 and dsRed2 were excited at 910 nm with 15 to100 mW of power delivered to the back-aperture of the objective. Green and red fluorescence signals were separated by a set of dichroic mirrors and filters (ET525/50m for green channel, ET629/56m for red channel). Image stacks were acquired at 512×512 pixels with a voxel size of 0.19 um in x and y with a z-step of 1 um. Representative images shown in figures were median filtered and contrast enhanced. To measure blood flow velocity, the mouse was intravenously injected with 20 ul Texas Red-dextran (10mg/ml, Invitrogen, USA). The motion of RBCs was identified from line-scan measurements, repetitive scans of the laser along the center axis of an arteriole (10-15 um in diameter).

### Two-photon targeted patching

We performed two-photon targeted patching as previously mentioned in the transfected mouse group [29, 47] and two-photon shadow patching in the control mouse group with no pre-labeled neurons according to the previous studies [48–50]. A patch pipette (6-8 MΩ) containing standard internal solution of the following composition: 112mM potassium gluconate, 8mM KCl, 10mM HEPES, 4mM Mg-ATP, 0.375mM Na2GTP, 10mM sodium phosphocreatine and 0.05mM Alexa594 for current-clamp; or 130mM CsMeSO3, 8mM NaCl, 10mM HEPES, 5mM QX314, 4mM Mg-ATP, 0.3mM Na-GTP, 0.2mM EGTA and 0.05mM Alexa 594 for voltage-clamp (pH7.3-7.4, 295mOsm). Recordings were done with an Axon patch 200B patch-clamp amplifier (Molecular Devices, Foster City, CA, USA) and Digidata 1550B interface (Molecular Devices, Foster City, CA, USA). Electrophysiological data were filtered at 10 kHz and sampled at 20 kHz. During the patching process, living image was obtained at 30 frames/sec with 512×512 pixel resolution. With the laser 910 nm wavelength, the electrode continued to advance to the fluorescent cells until attaching the soma of the cells (Supplementary Figure 3B top panel). Then turn the wavelength to 800 nm to see clear outline of the somata (Supplementary Figure 3B below panel). While control group, all procedure with 800 nm wavelength. After GΩ-seal formation, gentle suction was applied to break through the cell membrane to establish whole-cell configuration (Supplementary Figure 3C). The recording duration of each cells varied from 30 minutes to 1 hour. The series resistance was continuously monitored and the data were rejected if the resistance was higher than 30 MΩ. Action potential onset were measured at the point where the slope exceeded 50 mV/ms [51, 52].

### Western blot assays

Protein from the cerebral cortex tissue was extracted using RIPA buffer (Pierce) in accordance with the manufacturer’s recommendations. To detect the membrane protein, the Mem-PER™ Plus Membrane Protein Extraction Kit (Thermo) was used. The protein concentration was quantified using a BCA kit. Equal amounts of protein were separated with SDS–acrylamide gel and transferred into a PVDF membrane (Millipore). After blocking with nonfat milk, the membranes were incubated with anti-GluR2-Receptor antibody, anti-GluR1-Receptor antibody, anti-Beta Actin antibody, or pan-cadherin in blocking buffer overnight at 4°C temperature. The membrane was then incubated with horseradish peroxidase-conjugated secondary antibody diluted in blocking buffer at room temperature for 2 hours. The signal was determined by enhanced chemiluminescence (ECL). Expression levels of membrane proteins were normalized to pan-cadherin. Expression levels of total proteins were normalized to Beta Actin.

### Image analysis

Image processing of calcium imaging was carried out using custom-written scripts in MATLAB (MathWorks Corporation, Natick, MA) and FIJI software. All pixels within the cell-based regions of interest (ROIs) were averaged to yield a time course (ΔF/F) for each spine, which was further analyzed by GraphPad Prism 8.

All spine dynamics and intensity analysis were performed using custom written software in Igor Pro (WaveMetrics, Lake Oswego, Oregon). Spine within each time-point were visually identified and manually marked as a 3D point at their tip using the raw imaging stacks from the structural dsRed2 channel. Detailed steps for data analysis as described previously^2^, include spine dynamics and intensity of GluA1.

### Statistical analysis

Data distribution was assumed to be normal but was not formally tested. When possible we used Kolmogorov-Smirnov test or the non-parametric Mann-Whitney test. Otherwise, we assumed the data points have a normal distribution and used Student’s t-test or ANOVA with post hoc Bonferroni correction. All the tests were two-sided, the experimental results were expressed as Mean ± SEM, the level of P<0.05 was considered significant. All relevant methodological and statistical information are shown in Supplementary Table 1.

## Supplementary Material

Supplementary Figures

Supplementary Table 1
