# Supplementary material for: Transient ischemia-reperfusion induces cortical hyperactivity and AMPAR trafficking in the somatosensory cortex
Source: Aging (Albany NY). 2020 Mar 9;12(5):4299–321. doi: 10.18632/aging.102881 (PMC7093173; doi:10.18632/aging.102881)
Supplement: Supplementary Figures [file aging-12-102881-s002..pdf]

SUPPLEMENTARY FIGURES

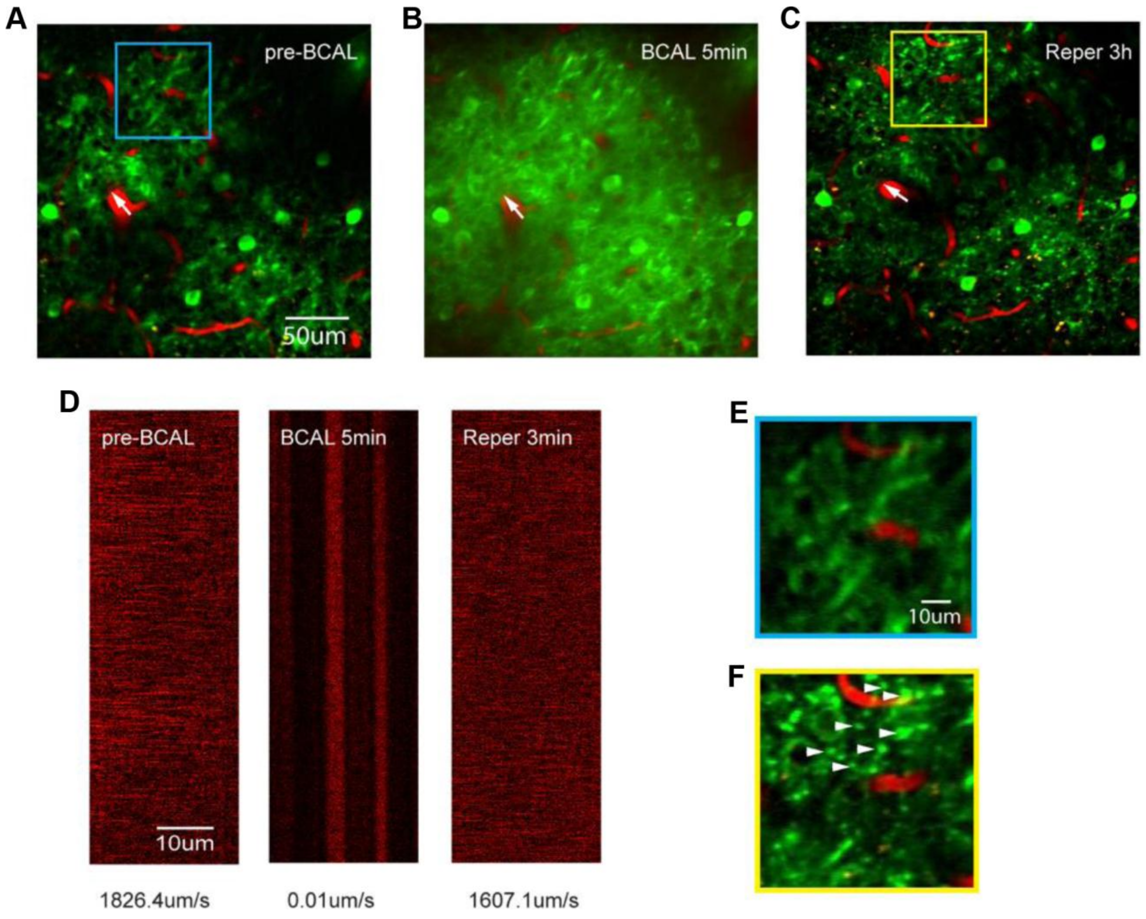

Supplementary Figure 1. Two-photon imaging *in vivo* to examine the effects of transient global ischemia on synaptic structures by ligating the bilateral common carotid arteries (**A**–**C**) Two-photon image showing Texas Red-dextran labeled blood vessels (red) and dendritic structures (green). (**D**) Two-photon line scanning images showing changes in blood flow velocity and flux in an arteriole labeled in (**A**–**C**) (white arrow). (**E**, **F**) Magnified view of the blue-boxed region in (**A**) and yellow-boxed region in (**C**) showing structural changes in dendritic spines after ischemia-reperfusion. Filled arrowhead indicates a stable spine on a narrow filament between dendritic beadings.

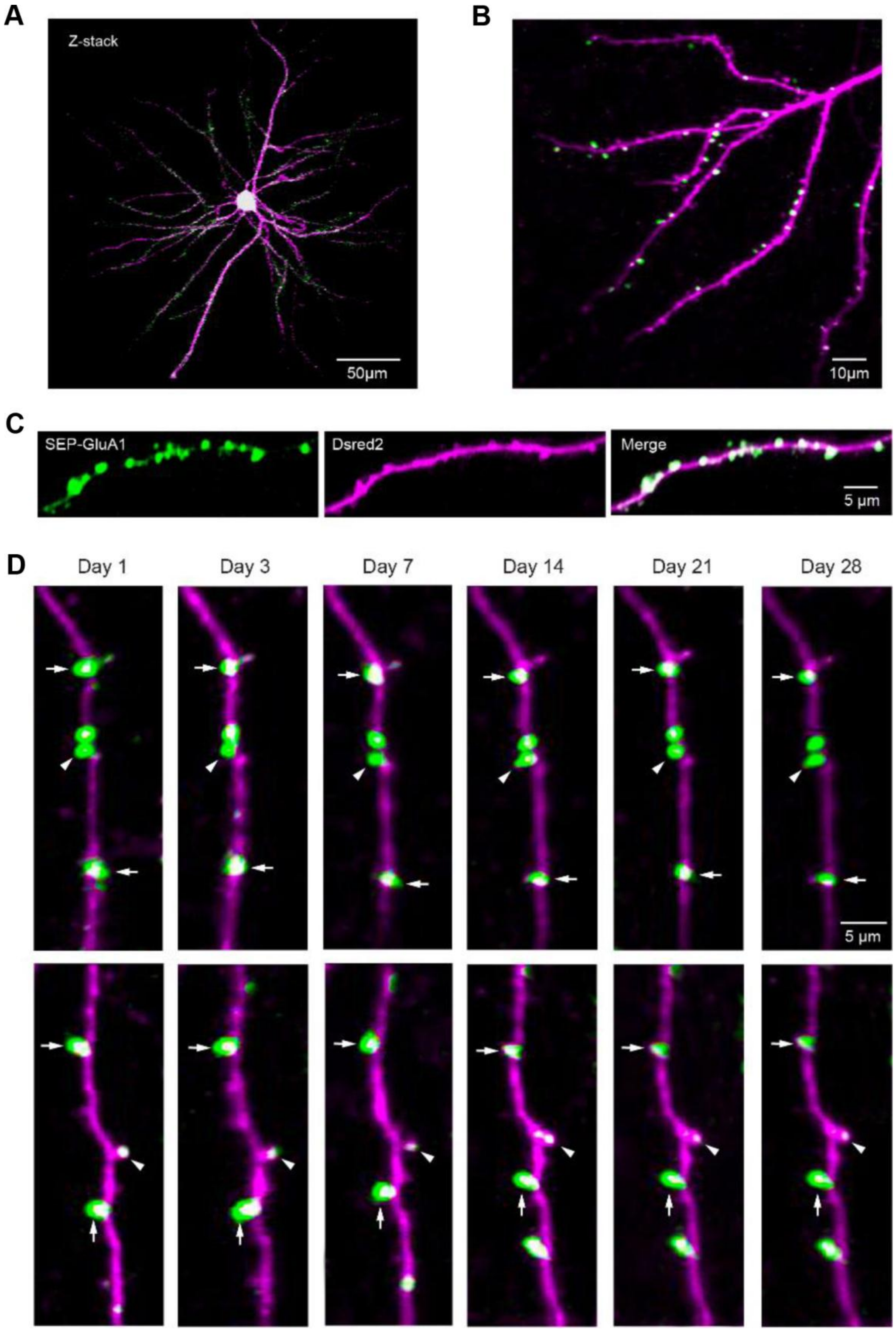

Supplementary Figure 2. Long-term expression stable of SEP-GluA1 in layer 2/3 somatosensory cortex neurons *in vivo*. (**A**) Z projection image of SEP-GluA1 tagged neuron. (**B**, **C**) Fluorescent images of SEP-GluA1 and dsRed2 in dendrites of layer 2/3 somatosensory cortex neurons. The RGB signal profile in (**C**) shows dsRed2 (magenta) and SEP-GluA1 (green) expression across the spine and dendritic shaft. (**D**) Long-term stable expression of SEP-GluA1 and dsRed2. Same spines were marked with arrows and arrowheads in different imaging sessions.

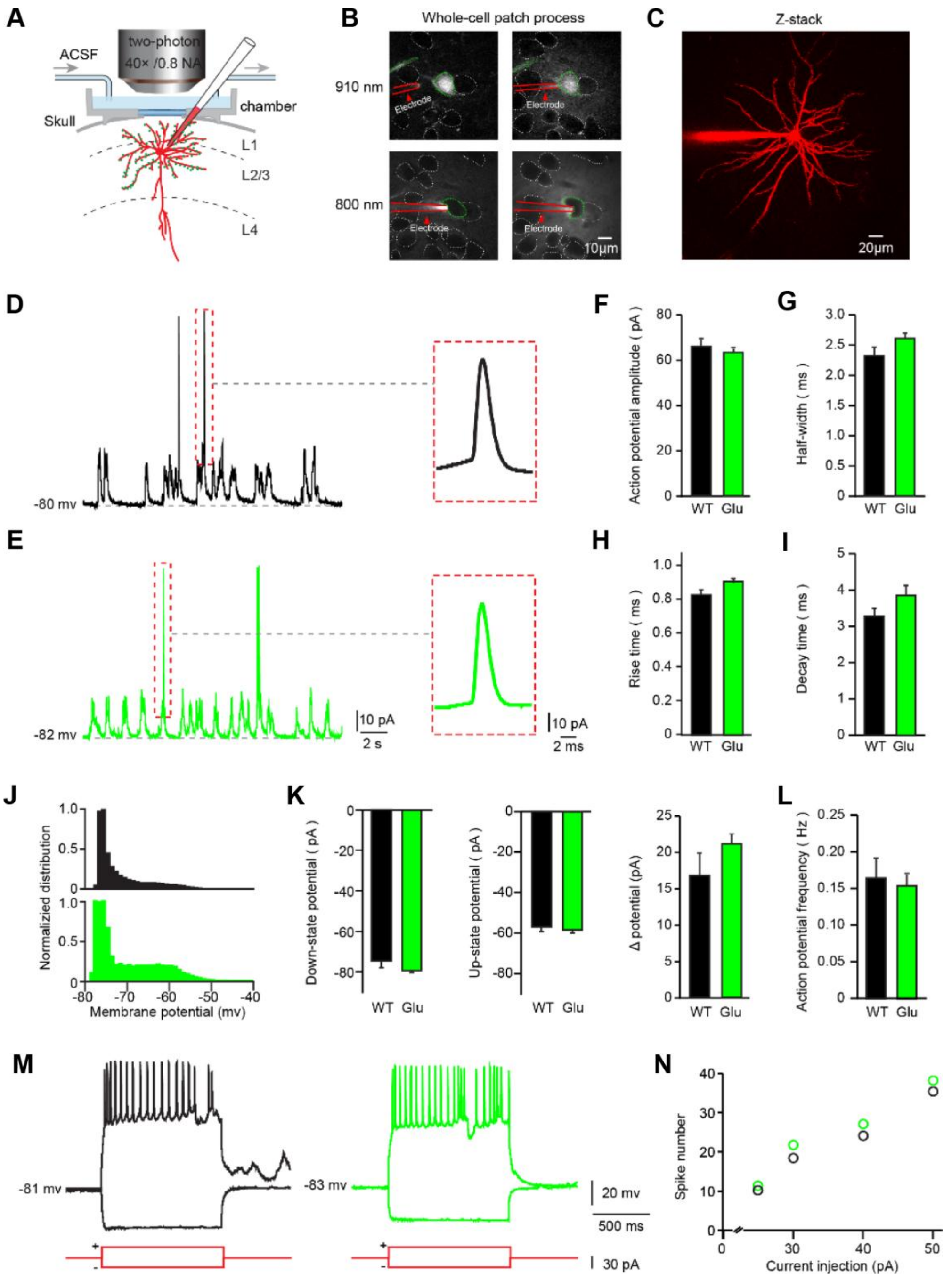

Supplementary Figure 3. Electrophysiological properties of two-photon targeted whole-cell recordings in somatosensory cortical neurons with sGluA1 *in vivo*. (**A**) View of the experimental setup. (**B**) Whole-cell configuration processing. Alexa594 is blown out from the electrode which was approaching to the tagged neurons, and can be seen with a 910 nm laser wavelength (the top two panels). For seeing the clear outline of the neurons, the wavelength was turned to 800 nm (the bottom two panels). The neurons are delineated with green dotted contours, and the electrode is denoted by a red arrowhead and is delineated with red lines. (**C**) Z-stack of in vivo two-photon images of a patched layer 2/3 (depth: 180 μm below the surface) pyramidal neuron. (**D**, **E**) Left panels, representative traces of membrane potential recorded from somatosensory cortex neurons in vivo. Right panels, magnification of the action potential (AP). (**F–I**) AP characteristics: AP amplitude. AP half-width. AP rise time. AP decay time. n= 6 neurons in 5 control mice, n= 6 neurons in 4 Glu mice. (**J**) Normalized distribution of membrane potential. (**K**) Left, down-state potential; middle, up-state potential and right, Δpotential. n= 4 neurons in 4 control mice, n= 6 neurons in 4 Glu mice. (**L**) AP frequency. n= 8 neurons in 5 control mice, n= 4 neurons in 4 Glu mice. (**M**) Intracellular current injection. (**N**) Spike number corresponds to different current intensities. ns, not significant, Student's t-test. Error bars = s.e.m.

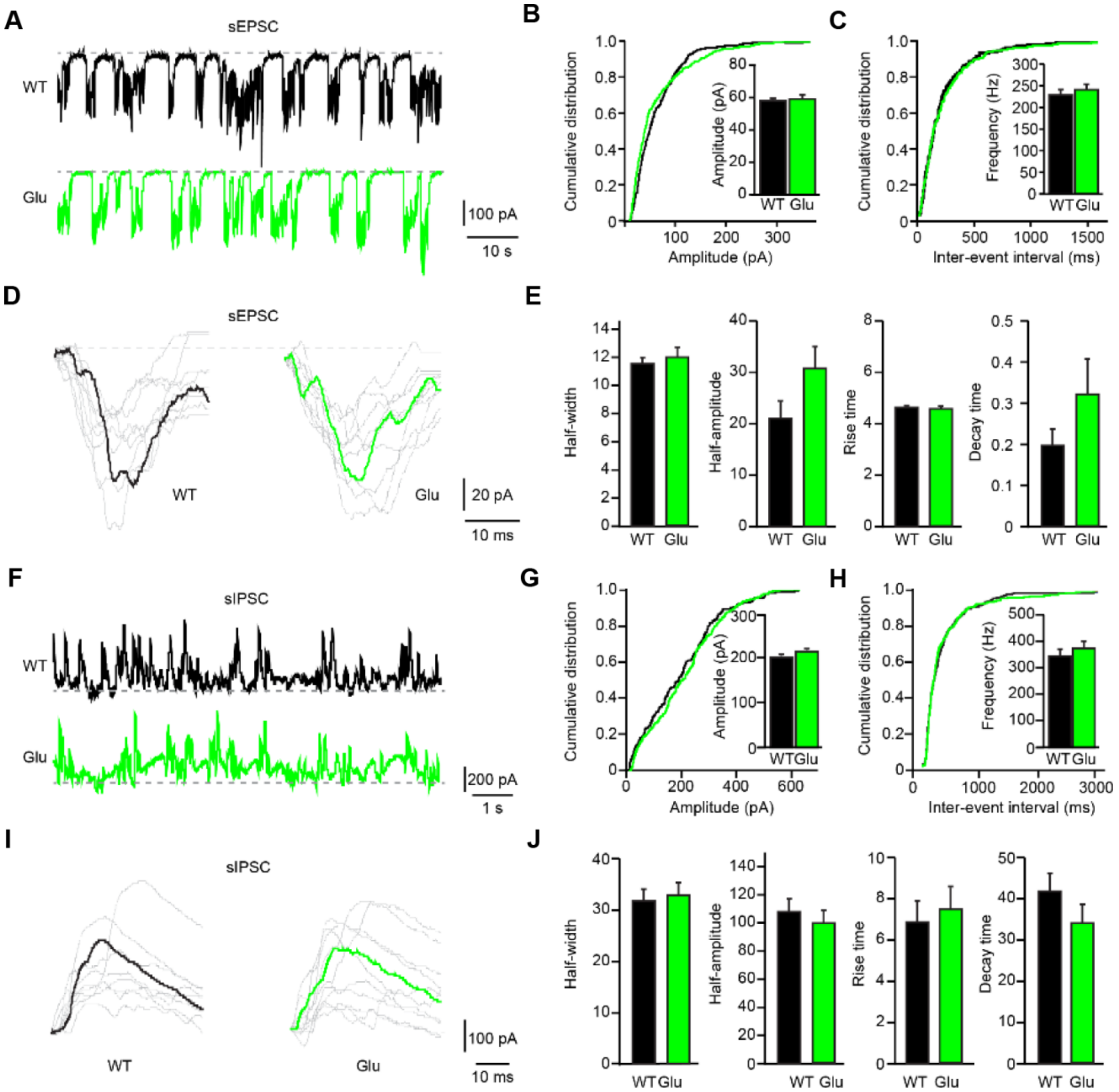

Supplementary Figure 4. Excitatory and inhibitory synaptic transmission features of cortical neurons with tagged AMPARs *in vivo*. (**A**) Representative traces of spontaneous EPSC (sEPSC) recorded from somatosensory cortex neurons *in vivo*. (**B**, **C**) Quantifications and cumulative distributions of sEPSC amplitudes and frequency. n= 8 neurons in 5 control mice and n= 6 neurons in 5 Glu mice. Kolmogorov-Smirnov test. (**D**) Representative traces of individual sEPSC. (**E**) sEPSC characteristics: half-width, half-amplitude, rise time, and decay time. Student's t-test. (**F**) Representative traces of spontaneous IPSC (sIPSC) recorded from somatosensory cortex neurons *in vivo*. (**G**, **H**) Quantifications and cumulative distributions of sIPSC amplitudes and frequency. n= 8 neurons in 5 control mice and n= 7 neurons in 4 Glu mice. Kolmogorov-Smirnov test. (**I**) Representative traces of individual sIPSC. (**J**) sIPSC characteristics: half-width, half-amplitude, rise time, and decay time. Student's t-test. ns, not significant, Error bars = s.e.m.

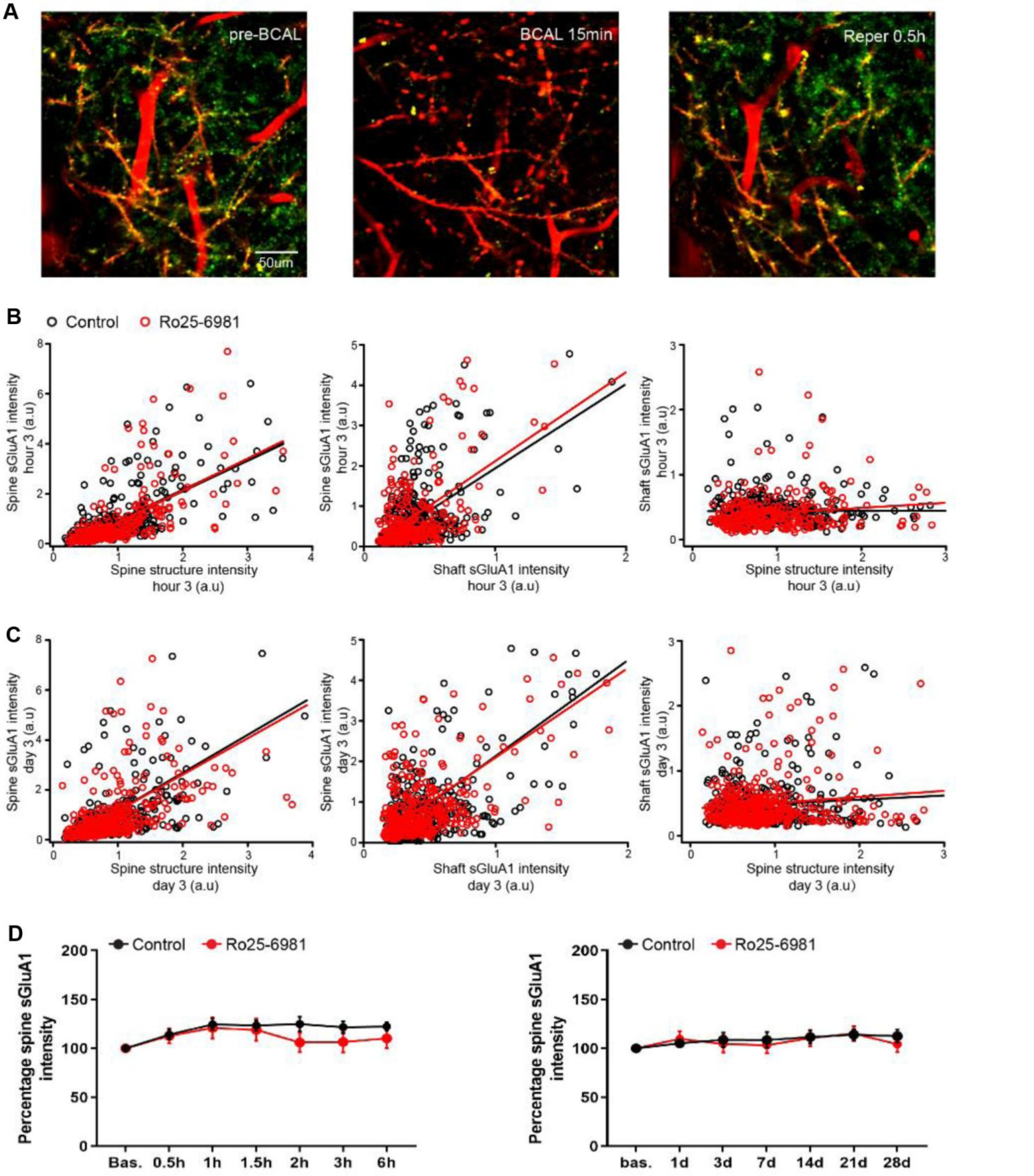

Supplementary Figure 5. No effect of NMDA receptor blockade on spine sGluA1 intensity over time in WT mice. (**A**) Two-photon image showing Texas Red-dextran labeled blood vessels (red), dendritic structures (red) and sGluA1 (green) (**B**) Correlation between spine sGluA1 intensity and spine structure intensity (left), spine sGluA1 and shaft sGluA1(middle), shaft sGluA1 and spine structure intensity (right) at the 3rd hour in the control and the treated mice. n= 410 spines in 5 control mice and n= 280 spines in 4 Ro25-6981-treated mice.(**C**) Correlation between spine sGluA1 and spine structure intensity (left), spine sGluA1 and shaft sGluA1(middle), and shaft sGluA1 and spine structure intensity (right) on the 3rd day in the control and the treated mice. n= 400 spines in 5 control mice and n= 405 spines in 4 Ro25-6981-treated mice. *r*, Pearson's linear correlation coefficient. *p*, Pearson's correlation t test. (**D**) NMDAR blockade showed no significant changes of Spine sGluA1 intensity (SEP-GluA1 signal) in the control (n= 5 mice) and the treated (n= 4 mice) mice over the first 6 hours (left) and 28 days (right) after reperfusion. Two-way ANOVA with Bonferroni correction. Error bars = s.e.m.

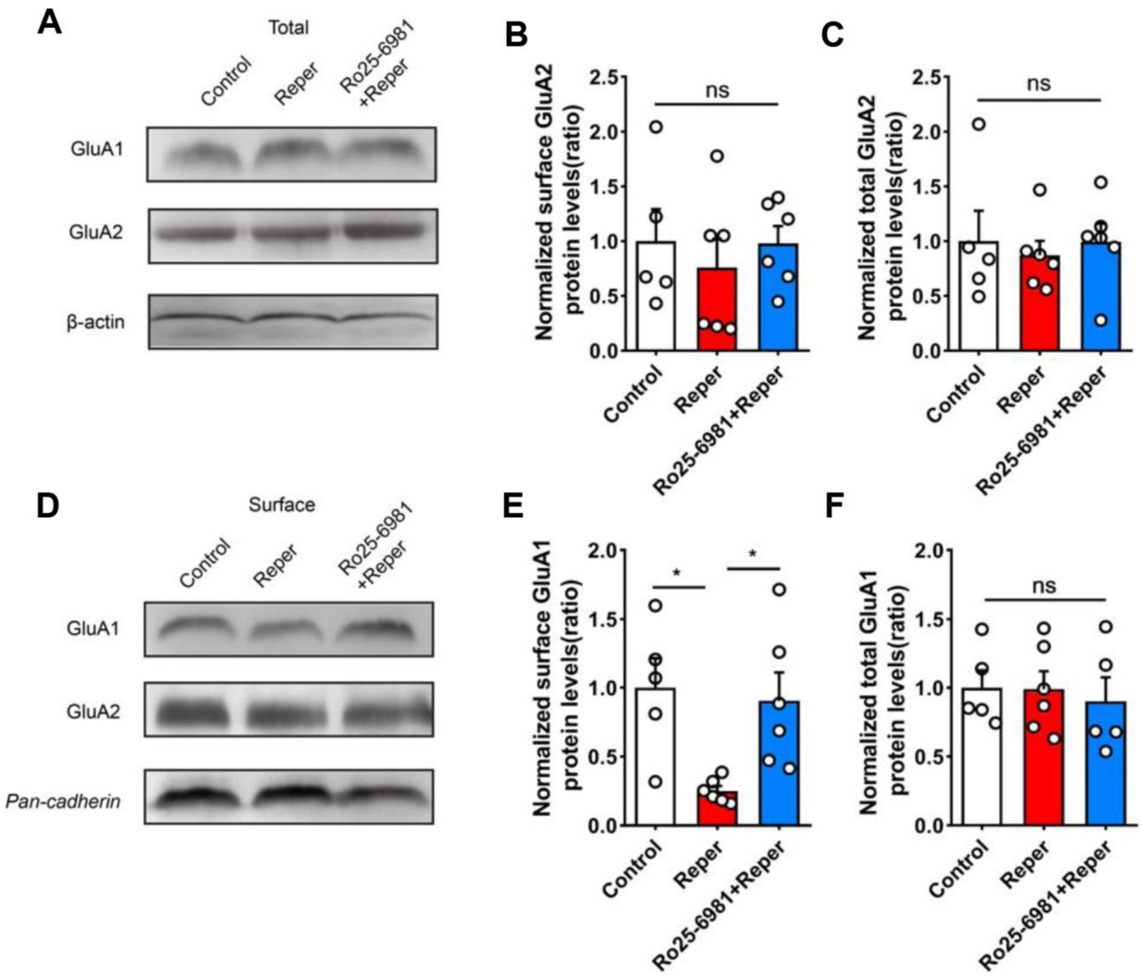

Supplementary Figure 6. Changes in AMPARs after transient global cerebral ischemia and NMDARs blockade. (**A**, **D**) Immunoblots of GluA1 and GluA2 from somatosensory cortex of control, Reper and Ro25-6981-treated mice. (**B**, **C**) Quantification of total GluA1 and GluA2 normalized to β-actin in the somatosensory cortex of control, Reper and Ro25-6981-treated mice. (**E**, **F**) Quantification of surface GluA1 and GluA2 normalized to Pan-cadherin in the somatosensory cortex of control, Reper and Ro25-6981-treated mice. n= 5 control mice, n= 6 reper mice, n= 6 Ro25-6981-treated mice. ns, not significant, *$p < 0.05$, one-way ANOVA with Bonferroni correction. Error bars = s.e.m.
