# Supplementary material for: Transient ischemia-reperfusion induces cortical hyperactivity and AMPAR trafficking in the somatosensory cortex
Source: Aging (Albany NY). 2020 Mar 9;12(5):4299–321. doi: 10.18632/aging.102881 (PMC7093173; doi:10.18632/aging.102881)
Supplement: Supplementary Table 1 [file aging-12-102881-s001..docx]

**Supplementary Table 1. Statistics reporting.**

|  | Test Used | n | | Descriptive Stats (Average, Variance) | P Value | Degrees of Freedom& F/t/z/R/ETC Value |
| --- | --- | --- | --- | --- | --- | --- |
| Figure Number | Which Test | Exat Value | Defined | Reported | Exat Value | Value |
| 1m (left) | unpaired t-test | 395 spines in control, 438 spines in Reper | 5 control animals, 6 Reper animals |  | p<0.001 | t=3.974 |
| 1m (middle) |  | 405 spines in control, 432 spines in Reper | 5 control animals, 6 Reper animals |  | p<0.001 | t=5.512 |
| 1m (right) |  | 397 spines in control, 436 spines in Reper | 5 control animals, 6 Reper animals |  | p=0.1937 | t=1.35 |
| 1n | unpaired t-test | 405 spines in control, 432 spines in Reper | 5 control animals, 6 Reper animals |  | p<0.001 | t=7.839 |
| 2g (left) | unpaired t-test | 395 spines in control, 312 spines in Ro25-6981+Reper | 5 control animals, 4 Ro25-6981+Reper animals |  | p=0.8475 | t=0.195 |
| 2g (middle) |  | 405 spines in control, 328 spines in Ro25-6981+Reper | 5 control animals, 4 Ro25-6981+Reper animals |  | p=0.4916 | t=0.7 |
| 2g (right) |  | 397 spines in control, 329 spines in Ro25-6981+Reper | 5 control animals, 4 Ro25-6981+Reper animals |  | p=0.205 | t=1.33 |
| 2h | unpaired t-test | 405 spines in control, 328 spines in Ro25-6981+Reper | 5 control animals, 4 Ro25-6981+Reper animals |  | p=0.103 | t=1.704 |
| 3c | two-way ANOVA | 1044 spines in Reper animals 824 spines in Ro25-6981+Reper animals | 6 Reper animals, 4 Ro25-6981+Reper animals | error bars are mean+/- SEM | time=3d: p=0.0045 | t=3.654 |
| 3d | two-way ANOVA | 1044 spines in Reper animals 824 spines in Ro25-6981+Reper animals | 6 Reper animals, 4 Ro25-6981+Reper animals | error bars are mean+/- SEM | all time: p>0.05 | N/A |
| 4i | Monte-Carlo shuffling | 1381 spines | 5 animals | r | p<0.001 | N/A |
| 4j | unpaired t-test | 27 neurons in control, 24 neurons in Glu | 5 control animals, 5 Glu animals | error bars are mean+/- SEM | p=0.149 | t=1.466 |
| 5d | one-way ANOVA | 425 spines in control, 379 spines in Reper, 411 spines in Ro25-6981+Reper | 5 control animals, 6 Reper animals, 5 Ro25-6981+Reper animals | error bars are mean+/- SEM | p=0.0015 p<0.01 | F=11.17 |
| 5f | one-way ANOVA | 410 spines in control, 283 spines in Reper, 274 spines in Ro25-6981+Reper | 5 control animals, 6 Reper animals, 5 Ro25-6981+Reper animals | error bars are mean+/- SEM | p=0.0647 p<0.01 | F=6.45 |
| 5g | one-way ANOVA | 400 spines in control, 215 spines in Reper, 208 spines in Ro25-6981+Reper | 5 control animals, 6 Reper animals, 5 Ro25-6981+Reper animals | error bars are mean+/- SEM | p>0.05 p>0.05 | F=1.67 |
| 6a (control) | Pearson's correlation t test | 342 | 5 animals | r | p<0.001 | r=0.72 slop value= 1.38 |
| 6a (BCAL) |  | 418 | 6 animals |  | p<0.001 | r=0.69 slop value=0.6 |
| 6a (BCAL+Ro25-6981) |  | 457 | 5 animals |  | p<0.001 | r=0.75 slop value=1.36 |
| 6b (control) | Pearson's correlation t test | 425 | 5 animals | r | p<0.001 | r=0.62 slop value=1.3 |
| 6b (BCAL) |  | 379 | 6 animals |  | p<0.001 | r=0.64 slop value=1.1 |
| 6b (BCAL+Ro25-6981) |  | 411 | 5 animals |  | p<0.001 | r=0.67 slop value=0.67 |
| 6c (control) | Pearson's correlation t test | 410 | 5 animals | r | p<0.001 | r=0.69 slop value=1.22 |
| 6c (BCAL) |  | 283 | 6 animals |  | p<0.001 | r=0.54 slop value=0.4 |
| 6c (BCAL+Ro25-6981) |  | 274 | 5 animals |  | p<0.001 | r=0.74 slop value=0.65 |
| 6d (control) | Pearson's correlation t test | 342 | 5 animals | r | p<0.001 | r=0.32 slop value= 1.4 |
| 6d (BCAL) |  | 418 | 6 animals |  | p<0.001 | r=0.31 slop value= 0.53 |
| 6d (BCAL+Ro25-6981) |  | 457 | 5 animals |  | p<0.001 | r=0.38 slop value= 1.82 |
| 6e (control) | Pearson's correlation t test | 425 | 5 animals | r | p<0.001 | r=0.46 slop value=1.9 |
| 6e (BCAL) |  | 379 | 6 animals |  | p<0.001 | r=0.44 slop value=1.73 |
| 6e (BCAL+Ro25-6981) |  | 411 | 5 animals |  | p<0.001 | r=0.40 slop value=1.41 |
| 6f (control) | Pearson's correlation t test | 410 | 5 animals | r | p<0.001 | r=0.37 slop value=1.47 |
| 6f (BCAL) |  | 283 | 6 animals |  | p<0.001 | r=0.31 slop value=0.51 |
| 6f (BCAL+Ro25-6981) |  | 274 | 5 animals |  | p<0.001 | r=0.36 slop value=1.02 |
| 6g (control) | Pearson's correlation t test | 342 | 5 animals | r | p=0.3046 | r=0.05 slop value= 0.02 |
| 6g (BCAL) |  | 418 | 6 animals |  | p=0.9359 | r=0.005 slop value= 0.003 |
| 6g (BCAL+Ro25-6981) |  | 457 | 5 animals |  | p=0.0985 | r=0.17 slop value= 0.07 |
| 6h (control) | Pearson's correlation t test | 425 | 5 animals | r | p=0.4060 | r=0.04 slop value=0.02 |
| 6h (BCAL) |  | 379 | 6 animals |  | p=0.8110 | r=0.012 slop value=0.005 |
| 6f(BCAL+Ro25-6981) |  | 411 | 5 animals |  | p=0.2526 | r=0.057 slop value=0.029 |
| 6i (control) | Pearson's correlation t test | 410 | 5 animals | r | p=0.9196 | r=0.005 slop value=0.002 |
| 6i (BCAL) |  | 283 | 6 animals |  | p=0.9866 | r=0.001 slop value=-0.001 |
| 6i (BCAL+Ro25-6981) |  | 274 | 5 animals |  | p=0.8301 | r=0.74 slop value=-0.004 |
| S 3e | unpaired t-test | 6 neurons in control animals, 6 neurons in Glu animals | 5 control animals, 4 Glu animals | error bars are mean+/- SEM | p=0.67 | t=0.4415 |
| S 3f | unpaired t-test | 6 neurons in control animals, 6 neurons in Glu animals | 5 control animals, 4 Glu animals | error bars are mean+/- SEM | p=0.11 | t=1.731 |
| S 3h | unpaired t-test | 6 neurons in control animals, 6 neurons in Glu animals | 5 control animals, 4 Glu animals | error bars are mean+/- SEM | p=0.13 | t=1.643 |
| S 3i | unpaired t-test | 6 neurons in control animals, 6 neurons in Glu animals | 5 control animals, 4 Glu animals | error bars are mean+/- SEM | p=0.09 | t=1.85 |
| S 3k | unpaired t-test | 4 neurons in control animals, 6 neurons in Glu animals | 4 control animals, 4 Glu animals | error bars are mean+/- SEM | Down-state potenital p=0.14 Down-state potenital p=0.46 potenital p=0.23 | t=1.642 t=0.783 t=1.286 |
| S 3l | unpaired t-test | 8 neurons in control animals, 4 neurons in Glu animals | 5 control animals, 4 Glu animals | error bars are mean+/- SEM | p=0.78 | t=0.2814 |
| S 4b | Kolmogorov-Smirnov test | 8 neurons in control, 6 neurons in Glu | 5 control animals, 5 Glu animals | error bars are mean+/- SEM | K-S=0.12 |  |
| S 4c | Kolmogorov-Smirnov test | 8 neurons in control, 6 neurons in Glu | 5 control animals, 5 Glu animals | error bars are mean+/- SEM | K-S=0.51 |  |
| S 4e | unpaired t-test | 8 neurons in control, 6 neurons in Glu | 5 control animals, 5 Glu animals | error bars are mean+/- SEM | Half-width p=0.48 Half-amplitude p=0.23 Raise time p=0.91 Decay time p=0.21 | t=0.7225 t=1.255 t=0.1164 t=1.321 |
| S 4g | Kolmogorov-Smirnov test | 8 neurons in control, 7 neurons in Glu | 5 control animals, 4 Glu animals | error bars are mean+/- SEM | K-S=0.25 |  |
| S 4h | Kolmogorov-Smirnov test | 8 neurons in control, 7 neurons in Glu | 5 control animals, 4 Glu animals | error bars are mean+/- SEM | K-S=0.54 |  |
| S 4j | unpaired t-test | 8 neurons in control, 7 neurons in Glu | 5 control animals, 4 Glu animals | error bars are mean+/- SEM | Half-width p=0.76 Half-amplitude p=0.57 Raise time p=0.71 Decay time p=0.31 | t=0.3179 t=0.5876 t=0.3762 t=1.046 |
| S 5b (control) | Pearson's correlation t test | 410 | 5 animals | r | p<0.001 | r=0.69 slop value=1.22 |
|  |  |  |  |  | p<0.001 | r=0.36 slop value=1.48 |
|  |  |  |  |  | p=0.9196 | r=0.005 slop value=0.002 |
| S 5b (Ro25-6981) | Pearson's correlation t test | 280 | 4 animal | r | p<0.001 | r=0.65 slop value=1.28 |
|  |  |  |  |  | p<0.001 | r=0.58 slop value=1.30 |
|  |  |  |  |  | p=0.0122 | r=0.14 slop value=0.07 |
| S 5c (control) | Pearson's correlation t test | 400 | 5 animals | r | p<0.001 | r=0.55 slop value=1.5 |
|  |  |  |  |  | p<0.001 | r=0.68 slop value=2.36 |
|  |  |  |  |  | p=0.0982 | r=0.08 slop value=0.065 |
| S 5c (Ro25-6981) | Pearson's correlation t test | 405 | 4 animals | r | p<0.001 | r=0.48 slop value=1.46 |
|  |  |  |  |  | p<0.001 | r=0.65 slop value=2.2 |
|  |  |  |  |  | p=0.0378 | r=0.1 slop value=0.093 |
| S 5d | two-way ANOVA |  | 5 control animals, 4 Ro25-6981 animals | error bars are mean+/- SEM | all time: p>0.05 | N/A |
| S 6b | one-way ANOVA |  | 5 control animals, 6 Reper animals, 5 Ro25-6981+Reper animals | error bars are mean+/- SEM | p=0.89 p=0.9 | F=0.15 |
| S 6c | one-way ANOVA |  | 5 control animals, 6 Reper animals, 6 Ro25-6981+Reper animals | error bars are mean+/- SEM | p=0.9 p=0.9 | F=0.14 |
| S 6e | one-way ANOVA |  | 5 control animals, 6 Reper animals, 6 Ro25-6981+Reper animals | error bars are mean+/- SEM | p<0.01 p<0.05 | F=8.01 |
| S 6f | one-way ANOVA |  | 5 control animals, 6 Reper animals, 6 Ro25-6981+Reper animals | error bars are mean+/- SEM | p=0.77 p=0.78 | F=0.32 |
